# Mechanisms of biliary stricture and novel therapeutic strategies

**DOI:** 10.3389/fmed.2025.1609461

**Published:** 2025-09-03

**Authors:** Boming Peng, Jianquan Zhang, Yang Xiang

**Affiliations:** ^1^Department of Hepatobiliary Surgery, Haikou Affiliated Hospital of Central South University Xiangya School of Medicine, Haikou, China; ^2^Haikou Key Laboratory of Clinical Research and Transformation of Digestive Diseases, Haikou, China

**Keywords:** biliary stricture, bile duct repair, targeted therapy, gene therapy, regenerative replacement therapy, tissue-engineered stents

## Abstract

Biliary stricture is a common yet complex pathological condition in clinical practice, which can be classified into iatrogenic and non-iatrogenic categories. Iatrogenic biliary strictures are mostly caused by cholecystectomy and hepatobiliary surgical procedures, while non-iatrogenic factors include immune-mediated, infectious, ischemic, genetic, idiopathic, and tumor-related causes. These factors induce damage to the biliary epithelium through various mechanisms, subsequently leading to bile stasis, inflammation, and fibrosis. The occurrence and progression of biliary strictures typically involve three major pathological processes: epithelial regeneration, inflammatory response, and fibrotic deposition. Following injury, the biliary epithelium can be repaired and maintained through mechanisms such as cholangiocyte proliferation, hepatocyte transdifferentiation, and activation of biliary stem/progenitor cells; meanwhile, the inflammatory response plays a dual role by promoting epithelial repair while also facilitating the progression of fibrosis. Multiple signaling pathways exert central regulatory roles throughout these processes. Currently, treatment strategies for biliary strictures have gradually evolved from traditional surgical interventions, such as hepaticojejunostomy and liver transplantation, to non-surgical approaches including immune modulation, bile acid metabolism regulation, and gut microbiota interventions. In recent years, novel therapeutic strategies for biliary strictures have emerged, including molecular therapies targeting fibrosis-related signaling pathways, gene-editing technologies, regenerative replacement therapies, and the development of advanced tissue-engineered scaffolds. These emerging approaches are expected to offer more efficient and safer solutions for the precision treatment of biliary strictures in the future.

## 1 Introduction

The biliary system plays a vital role in digestion and bile transport. As the main cellular component of the biliary system, cholangiocytes are responsible for absorption, secretion, and the regulation of bile (1). Once biliary epithelial cells are injured, structural and functional abnormalities may occur, leading to biliary stricture, cholestasis, and severe hepatic disorders. Biliary stricture is not only a central pathological feature of various biliary and hepatobiliary diseases but also an important clinical cause of jaundice, cholestatic cirrhosis, and even liver failure (2). The etiology of biliary stricture is complex, including iatrogenic factors such as surgical procedures, as well as immune-mediated, infectious, vascular and ischemic, genetic, idiopathic, and tumor-related causes (3). Each etiology has distinct characteristics regarding the affected sites, disease features, and rate of progression. From a pathological perspective, biliary stricture is not a single event but rather a continuous yet overlapping triphasic response following bile duct injury: epithelial regeneration, inflammatory regulation, and fibrotic deposition. Among these, the inflammatory response exerts critical regulatory roles via multiple signaling pathways, providing essential signals for epithelial regeneration while simultaneously promoting the progression of fibrosis.

The treatment paradigm for biliary stricture is shifting from a surgery-centered approach aimed primarily at relieving mechanical obstruction to a stratified, mechanism-based, and regeneration-oriented comprehensive management model. Traditional surgical procedures (hepaticojejunostomy, hepatic resection, and liver transplantation) remain essential for the treatment of severe or complex strictures, but postoperative complications and anatomical limitations have driven the search for alternative strategies. Non-surgical interventions, such as immune modulation, bile acid regulation, and gut microbiota modulation, are being applied in different etiological contexts to control inflammation and delay fibrosis. Meanwhile, emerging therapeutic platforms are rapidly evolving: molecular targeted agents against fibrogenic pathways, gene-editing techniques for correcting genetic defects, regenerative replacement therapies, and tissue-engineered scaffolds combined with endoscopic interventions (e.g., ERCP) for reconstructing biliary continuity–all of which show translational potential.

This review is structured around the framework of “etiology–pathological stages–therapeutic strategies.” First, we summarize the major etiologies of biliary stricture and their intrahepatic and extrahepatic involvement characteristics. Next, we systematically examine the cellular and molecular mechanisms underlying the three key reparative axes–epithelial regeneration, inflammatory response, and fibrotic deposition. Finally, we review current surgical and non-surgical management strategies, with particular emphasis on the progress and clinical prospects of emerging approaches such as targeted therapy, gene and regenerative medicine, and tissue-engineered scaffolds, aiming to provide insights for the precision diagnosis, treatment, and future research of biliary stricture.

## 2 Types of biliary stricture

The causes of biliary stricture can be broadly categorized into iatrogenic and non-iatrogenic factors. Non-iatrogenic injuries include immune-mediated, infectious, vascular and ischemic, genetic, idiopathic, and tumor-related damage. These factors can lead to bile outflow obstruction and cholestasis, exacerbating bile duct injury and triggering a cascade of pathological responses.

### 2.1 Iatrogenic biliary stricture

Iatrogenic biliary stricture is primarily caused by surgical procedures and medications, with surgery mainly leading to extrahepatic bile duct injury and stricture, while medications predominantly affect the intrahepatic bile ducts. Cholecystectomy is the most common cause of iatrogenic biliary stricture. The mechanism of surgery-related biliary stricture is mainly due to mechanical injury during the operation (4). In addition, postoperative benign biliary strictures can be exacerbated by bile stasis and recurrent cholangitis, further aggravating biliary injury and narrowing. Drug-induced biliary stricture is pathologically characterized by cholangitis-like lesions, accompanied by biliary epithelial cell damage and cholestasis. During the use of chemotherapeutic agents and immune checkpoint inhibitors, secondary sclerosing cholangitis is frequently observed (5, 6).

### 2.2 Non-iatrogenic biliary stricture

#### 2.2.1 Immune-mediated stricture

Primary biliary cholangitis (PBC), primary sclerosing cholangitis (PSC), and IgG4-related cholangitis (IgG4-RC) are all immune-mediated biliary strictures, but their pathological features, immune injury mechanisms, and sites of involvement vary. PBC primarily affects the intrahepatic bile ducts and is a chronic autoimmune biliary disease characterized by immune-mediated destruction of interlobular bile ducts, often accompanied by positive antimitochondrial antibodies, which gradually lead to cholestasis and liver fibrosis. Studies have shown that abnormal B cell activation and Th17 immune responses play important roles in the pathogenesis of PBC (7). PSC is a chronic inflammatory biliary disease that involves both intrahepatic and extrahepatic bile ducts, characterized by bile duct fibrosis and strictures. It is frequently associated with inflammatory bowel disease, suggesting the importance of intestinal immune dysregulation in its pathogenesis (8). During the course of PBC and PSC, Kupffer cells play an important role in regulating local immune balance by secreting pro-inflammatory and anti-inflammatory factors (9). Furthermore, patients with these conditions often exhibit decreased expression levels of TGR5, a change that not only suppresses bile flow but also promotes cholangiocyte apoptosis, thereby aggravating biliary injury (10). IgG4-RC also involves abnormal activation of immune cells, characterized by massive infiltration of IgG4-positive plasma cells and fibrosis, and is closely associated with abnormal activation of regulatory T cells (Treg) and related cytokines (11).

#### 2.2.2 Infectious Stricture

Pathogens that induce infectious biliary strictures include bacteria, parasites, and viruses, which mediate bile duct injury and stricture formation through complex pathological mechanisms. Generally, bacterial infections primarily affect the extrahepatic bile ducts, whereas parasitic and viral infections are more often associated with intrahepatic bile duct injury and stricture. Bacterial infections typically originate from the intestine. When bacteria invade the biliary tract, antigen-presenting cells recognize and present bacterial antigens, inducing immune cells to release pro-inflammatory factors and activate local inflammatory responses (12). Moreover, gut microbiota dysbiosis plays an important role in infection-related biliary injury: microbial imbalance not only affects the hydrophobicity and cytotoxicity of bile acids by altering enzyme activity but also disrupts the intestinal barrier, allowing microbes and their metabolites to enter the liver via the portal vein, thereby triggering biliary inflammation and fibrosis (13, 14). Parasitic infections induce stricture formation by causing physical injury or releasing chemical stimuli that continuously provoke inflammatory responses in the biliary epithelium (15). In this process, inflammatory cells (particularly T cells) and gut microbiota jointly regulate inflammation and tissue injury (16). Additionally, viral infections (such as human immunodeficiency virus, cytomegalovirus, and SARS-CoV-2) have also been implicated in the development of biliary fibrosis, although their exact mechanisms remain to be elucidated (17, 18).

#### 2.2.3 Vascular and ischemic stricture

Ischemic biliary stricture can be induced by various factors, including intraoperative vascular clamping and injury, arterial obstruction caused by medications or interventional hemostasis, as well as systemic hypoperfusion states associated with conditions such as AIDS and shock, all of which ultimately lead to ischemic injury of intrahepatic bile ducts and subsequent stricture formation. The blood supply to the biliary tract mainly depends on the peribiliary plexus (PBP), formed by branches of the proper hepatic artery. The close anatomical relationship between the PBP and biliary epithelial cells makes this area particularly susceptible to ischemia while also providing a direct pathway for functional regulation between them. Consequently, the PBP not only maintains the metabolic and secretory functions of cholangiocytes but is also closely associated with the pathogenesis of various biliary diseases.

Following liver transplantation, the two major pathological bases for biliary injury and stricture are ischemia-reperfusion injury and hepatic artery thrombosis. Hepatic artery thrombosis can lead to insufficient blood flow to the PBP and impair biliary epithelial regeneration (19). Ischemia-reperfusion injury, on the other hand, is a major mechanism underlying non-anastomotic biliary strictures after liver transplantation, often accompanied by cholestasis and cholangitis (20). Additionally, cholangiocytes are highly sensitive to ischemia; while mild and transient ischemia may allow re-epithelialization and partial functional recovery, sustained or severe ischemia promotes fibrosis progression and leads to biliary stricture. Similarly, hepatic arterial ischemia can suppress the function of hepatocellular bile transporters, aggravating cholestasis and further damaging the structure of the gallbladder and bile ducts, forming a vicious cycle of injury, stasis, and fibrosis (21).

#### 2.2.4 Genetic stricture

Alagille syndrome and cystic fibrosis (CF) are biliary stricture disorders caused by genetic factors, primarily affecting the intrahepatic bile ducts. The pathological hallmark of Alagille syndrome is a reduction in intrahepatic interlobular bile ducts, and its pathogenesis is closely associated with mutations in the Jagged1 or Notch2 genes. These mutations disrupt the critical regulatory role of the Notch signaling pathway in bile duct differentiation and development, leading to abnormal bile duct formation and intrahepatic cholestasis. In addition to hepatic manifestations, Alagille syndrome is frequently accompanied by cardiovascular, skeletal, and ocular abnormalities, indicating that it is a multisystem developmental disorder (22).

Cystic fibrosis liver disease is a complex biliary disorder characterized by CFTR dysfunction–induced bile acid imbalance, bile stasis, inflammatory responses, and bile duct injury. It is closely associated with disruptions in the gut-liver axis and intestinal dysbiosis, which together activate inflammatory and fibrotic pathways, thereby promoting progression of hepatobiliary injury. Patients with cystic fibrosis often present with multi-organ involvement, including the pancreas and respiratory system. Hepatobiliary manifestations may become apparent during childhood and can gradually progress to biliary cirrhosis (23).

#### 2.2.5 Idiopathic stricture

Among idiopathic biliary strictures, biliary atresia (BA) is the most representative condition. It is characterized by progressive fibrosis and obliteration of the bile ducts, leading to impaired bile drainage and progressive liver injury, and is one of the most common causes of neonatal cholestasis (24). Studies have shown that in BA, biliary injury predominantly occurs in the extrahepatic bile ducts and is primarily driven by T cell–mediated immune responses. In particular, Th1 and Treg cells regulate the IFN-γ/STAT signaling pathway to induce biliary fibrosis, significantly influencing disease progression (25). Furthermore, increased expression of YAP1 is another important factor contributing to BA progression, as it enhances pathological proliferation of biliary cells and activates pro-fibrotic pathways in hepatic stellate cells, further promoting biliary structural damage and fibrosis (26). Overall, the pathogenesis of BA is highly complex, with the interplay of multiple signaling pathways jointly accelerating biliary stricture formation and disease progression.

#### 2.2.6 Tumor-associated injury

Cholangiocarcinoma is a rare but highly aggressive malignant tumor originating from biliary epithelial cells. Based on its anatomical location, it can be classified into intrahepatic, perihilar, and distal types. Recent studies have demonstrated that the development of cholangiocarcinoma is not only closely associated with genetic mutations and exposure to carcinogens but is also inseparably linked to chronic biliary inflammation and cholestasis (27). These pathological conditions continuously stimulate biliary epithelial cells, leading to uncontrolled cell proliferation, impaired DNA damage repair, and aberrant epigenetic modifications, thereby promoting malignant transformation. At the molecular level, signaling pathways such as MAPK/ERK, PI3K/AKT, and Wnt/β-catenin play critical roles in regulating cholangiocarcinoma cell proliferation, inhibition of apoptosis, invasion, and the development of therapeutic resistance (28).

### 2.3 Secondary biliary injury

Biliary strictures caused by various etiologies often present as biliary obstruction and cholestasis, which further exacerbate biliary injury and fibrosis through multiple mechanisms. In this process, immune cells–particularly macrophages–play a key role by modulating immune-inflammatory responses (29). Biliary epithelial cells interact with stromal cells through autocrine and paracrine mechanisms, stimulating their own proliferation and, under conditions of chronic inflammation, potentially driving the onset of cholangiocarcinoma (30). Furthermore, studies have revealed that signaling pathways such as Notch, Wnt, and TGF-β play central regulatory roles in the pathogenesis and progression of secondary cholestatic biliary stricture. By activating the expression of genes associated with biliary epithelial proliferation and fibrosis, these pathways collectively drive the pathological process (31–33) (Table 1).

**TABLE 1:** Causes and treatment strategies of biliary tract stenosis.

Etiology		Primary affected region	Treatment approaches
Iatrogenic	Surgical	Extrahepatic bile ducts (4)	Surgical treatment, tissue-engineered stents
	Pharmacological	Intrahepatic bile ducts (34)	Gene therapy
Immune-Mediated	Primary biliary cholangitis	ntrahepatic bile ducts (35)	Immunomodulation, gut microbiota modulation
	Primary sclerosing cholangitis	Intra- and extrahepatic bile ducts (36)	Immunomodulation, gut microbiota modulation
	IgG4-related cholangitis	Intra- and extrahepatic bile ducts (37)	Immunomodulation, gut microbiota modulation
Infectious	Bacterial	Extrahepatic bile ducts (38)	Gut microbiota modulation
	Parasitic	Intrahepatic bile ducts (39)	–
	Viral	Intrahepatic bile ducts (40)	–
Vascular or ischemic	Local arterial occlusion	Intrahepatic bile ducts (41)	Surgical treatment, organoids
	Systemic hypoperfusion	Intrahepatic bile ducts (42)	–
Genetic	Alagille syndrome	Intrahepatic bile ducts (43)	Gene therapy
	Cystic fibrosis	Intrahepatic bile ducts (44)	Gene therapy
Idiopathic	Biliary atresia	Extrahepatic bile ducts (45)	Surgical treatment, organoids
Tumor-Associated	Cholangiocarcinoma	Intra- and extrahepatic bile ducts (46)	Surgical treatment, immunomodulation, gene therapy,
Secondary cholestasis	–	Intra- and extrahepatic bile ducts	Bile acid modulation, targeted therapy

## 3 Mechanisms of biliary stricture

Anatomically, the biliary system is divided into intrahepatic and extrahepatic bile ducts, which differ significantly in their embryonic origins and differentiation pathways. Intrahepatic bile duct cells originate from hepatoblasts located near the portal vein and surrounding mesenchyme, whereas extrahepatic bile duct cells are derived directly from the endoderm. Bile ducts can also be classified by diameter into small bile ducts (*D* < 300 μm) and large bile ducts (*D* > 300 μm), which differ in morphology, function, and injury repair mechanisms (1). However, regardless of whether the bile duct cells are intrahepatic or extrahepatic, they generally undergo three pathological stages following injury: epithelial regeneration and repair, inflammatory Response, and fibrotic Deposition. These processes involve autocrine and paracrine regulatory mechanisms of biliary epithelial cells, coordinated by growth factors, cytokines, neurotransmitters, and hormones, all of which play key roles at different stages of repair (47). Although the etiologies of biliary stricture are complex, bile duct cell injury typically proceeds through similar pathological phases. Elucidating the mechanisms of stricture from the novel perspective of these post-injury pathological stages not only deepens our understanding of the dynamic repair processes but may also provide a theoretical basis for the development of novel therapeutic strategies for biliary strictures.

### 3.1 Epithelial regeneration

The ductular reaction (DR) represents the regenerative repair process of the biliary system in response to injury, characterized by the reactive proliferation of biliary epithelial cells. Following biliary epithelial injury, four mechanisms contribute to maintaining epithelial integrity: proliferation of biliary epithelial cells; transdifferentiation of hepatocytes into biliary epithelial cells; proliferation and differentiation of hepatic progenitor cells (HPC); and activation and differentiation of biliary tree stem/progenitor cells (BTSCs). Under normal conditions, most biliary epithelial cells remain in a quiescent state (G0 phase) but retain a certain regenerative potential. In the case of mild injury, damaged biliary epithelium repairs itself by releasing cytokines that induce proliferation of neighboring healthy biliary epithelial cells. In chronic or severe injury, hepatocytes can transdifferentiate into biliary epithelial cells to promote repair and regeneration. In this process, IL-6 secreted by Kupffer cells plays a stimulatory role (48), under the precise regulation of signaling pathways such as Notch, TGF-β, and Wnt (49).

When the severity of injury exceeds the proliferative capacity of biliary epithelial cells, HPCs located in the canals of Hering and peribiliary glands (PBGs) become activated. They proliferate and differentiate into biliary epithelial cells to preserve biliary integrity, a process that is also regulated by Wnt and Notch signaling pathways (50). For damage involving extrahepatic bile ducts or larger intrahepatic ducts, BTSCs within PBGs represent the primary source of reparative cells (51).

Moreover, bile duct repair is not solely dependent on biliary epithelial cells but is also influenced by inflammatory cells and stromal cells. The inflammatory microenvironment promotes biliary epithelial cell regeneration and migration through the secretion of growth factors, cytokines, and matrix remodeling factors. It is noteworthy that there is a delicate balance between the intensity of inflammation and the repair response. When inflammation persists or becomes excessive, the DR transitions from a regenerative to a pathological state, ultimately driving fibrosis and structural abnormalities of the biliary tract.

### 3.2 Inflammatory response

Simultaneously with the regeneration of biliary epithelium following injury, a local inflammatory response is promptly triggered. Interestingly, in certain biliary diseases, dysregulated, excessive, or persistent activation of inflammatory cells becomes a key factor driving the progression of biliary injury. The inflammatory response phase involves the dynamic participation of multiple cell types, among which biliary epithelial cells are not only activated but also modulate the local immune microenvironment via autocrine and paracrine mechanisms, contributing to the immune-inflammatory response of biliary injury. Neutrophils and monocytes are typically the first inflammatory cells recruited to the injury site. Through the release of cytokines and chemokines, they participate in amplifying the inflammatory cascade while also providing critical signals for tissue repair (52).

Bile stasis resulting from biliary injury is considered a key physical and chemical stimulus that can regulate immune cell activation, differentiation, and cytokine secretion by activating nuclear receptor signaling pathways such as FXR and VDR. In addition, accumulated bile acids act as endocrine signaling molecules, further modulating local inflammatory responses, epithelial apoptosis, and fibrotic progression through bile acid signaling pathways such as FXR and TGR5 (53). Moreover, studies have shown that innate immune receptors, such as TLRs, play a central role in sensing biliary injury and infectious agents, enhancing the release of pro-inflammatory factors via activation of downstream signaling pathways such as NF-κB (54). In contrast, PPARs play a negative regulatory role in maintaining biliary metabolic homeostasis and suppressing inflammatory responses, attenuating excessive inflammation through transcriptional repression mechanisms (55). The Hippo signaling pathway regulates YAP/TAZ activity, influencing biliary epithelial cell proliferation and repair, while its dysregulation is closely associated with abnormal biliary epithelial cell proliferation and fibrosis (56). The Hedgehog signaling pathway mediates epithelial-mesenchymal crosstalk, regulating the DR and fibroblast activation, thereby exerting a dual regulatory effect on the balance between inflammation and repair after biliary injury (57).

Multiple inflammation-related signaling pathways are involved in the inflammatory repair process following biliary injury. However, how to precisely control the inflammatory response–ensuring it promotes tissue repair without tipping toward excessive inflammation or fibrosis–has become a critical challenge in the treatment of biliary injury and warrants further in-depth investigation.

### 3.3 Fibrotic deposition

Chronic biliary injury often leads to a pathological repair response characterized by excessive deposition and abnormal distribution of extracellular matrix (ECM), ultimately resulting in fibrosis and irreversible structural changes of the biliary tract. This process is orchestrated by multiple cell populations and signaling pathways. During fibrotic repair, activated biliary epithelial cells not only recruit inflammatory and stromal cells (particularly hepatic stellate cells, HSCs) through the upregulation of cytokine and chemokine secretion, but also, through direct or indirect crosstalk with these cells, promote ECM synthesis and deposition, thereby driving the initiation and progression of fibrosis.

Furthermore, dysbiosis of the gut microbiota is recognized as an important contributing factor to biliary fibrosis. It can disrupt biliary epithelium integrity and trigger chronic inflammation-mediated fibrogenesis (38). Several key signaling pathways play crucial roles in this process. Studies have shown that the TGF-β signaling pathway is a central driver of fibrosis through activation of Smad-dependent mechanisms, which promote HSC activation (58). The Wnt/β-catenin signaling pathway regulates biliary epithelial cell proliferation and epithelial-mesenchymal transition (EMT), and it synergizes with TGF-β in promoting fibrosis (59). The Notch signaling pathway regulates the differentiation state of HPCs and BTSCs, thus exerting essential control over the balance between biliary regeneration and fibrosis (60). In addition, the PI3K/AKT signaling pathway contributes to cell survival and ECM regulation, while the TLR signaling pathway mediates the interplay between immune-inflammatory responses and fibrogenesis (61, 62). The biliary fibrotic repair process exhibits a double-edged sword effect: on one hand, excessive ECM deposition inhibits normal epithelial regeneration, leading to complications such as biliary stricture and bile stasis; on the other hand, early fibrotic signaling also provides the necessary microenvironment and structural support for epithelial regeneration.

Balancing the prevention of excessive fibrosis with the promotion of effective regeneration remains a major challenge in the treatment of biliary injury. Future studies should focus on elucidating the dynamic network interactions among different signaling pathways and exploring multi-target combinational interventions to simultaneously modulate fibrosis and promote epithelial repair, thereby providing a more precise theoretical basis for clinical treatment (Table 2).

**TABLE 2:** Signal pathways involved in the pathological process of biliary stenosis and their functions.

Pathological process	Signal pathways	Functions
Epithelial regeneration	Notch	Hepatocyte transdifferentiation; HPC proliferation and differentiation
	TGF-β	Hepatocyte transdifferentiation
	Wnt	Hepatic progenitor cell proliferation and differentiation
Inflammatory response	FXR	Immune cell activation, differentiation, and cytokine secretion; regulation of fibrosis progression
	VDR	Immune cell activation, differentiation, and cytokine secretion
	TGR5	Regulation of local inflammatory responses, epithelial apoptosis, and fibrosis progression
	TLR	Activation of downstream inflammatory pathways such as NF-κB to enhance inflammatory cytokine release
	PPAR	Negative regulatory role in maintaining biliary metabolic homeostasis and inhibiting inflammatory responses, mitigating excessive inflammation through transcriptional repression
	Hippo	Regulation of biliary epithelial cell proliferation and repair via modulation of YAP/TAZ activity; dysregulation closely associated with abnormal cholangiocyte proliferation and fibrosis
	Hedgehog	Participation in epithelial-mesenchymal crosstalk, regulation of ductular reaction and fibroblast activation, playing a dual role in balancing inflammation and repair after biliary injury
Fibrotic deposition	TGF-β	Promotion of hepatic stellate cell (HSC) activation via Smad-dependent signaling pathway, acting as a core driver of fibrosis formation
	Wnt	Regulation of biliary epithelial cell proliferation and epithelial-mesenchymal transition (EMT), synergizing with TGF-β signaling
	Notch	Modulation of differentiation status of HPCs and biliary tree stem/progenitor cells (BTSCs), critically regulating the balance between biliary regeneration and fibrosis
	PI3K/AKT	Involvement in cell survival and extracellular matrix (ECM) regulation
	TLR	Mediation of the interaction between immune-inflammatory responses and fibrosis

## 4 Treatment of biliary stricture and injury

Different types of biliary injuries require treatment strategies with distinct focal points. Targeted therapy based on the underlying etiology and pathological mechanisms not only improves the efficiency of repair but also aligns with the current medical paradigm of personalized treatment. At present, treatment approaches for biliary stricture and injury include traditional surgical procedures, non-surgical interventions, and emerging cutting-edge therapies, providing patients with a wide range of therapeutic options.

### 4.1 Surgical treatment

The primary goals of surgical treatment for biliary stricture are to remove lesions, reconstruct the bile duct, and reduce the risk of post-injury complications. Surgical procedures are commonly applied in iatrogenic and malignant biliary strictures. Conventional surgical interventions for bile duct injuries include hepaticojejunal anastomosis (HJ), percutaneous transhepatic biliary drainage (PTBD), hepatectomy, and liver transplantation (LT).

For intrahepatic biliary strictures, hepatectomy may serve as an emergency intervention for acute, localized, and severe biliary injury. However, postoperative complications such as bile leakage and infection must be carefully monitored (63). In cases of severe or diffuse intrahepatic strictures, particularly when persistent cholestasis progresses to liver failure, LT–despite being a high-risk procedure (64)–remains the only effective treatment option. Common post-LT complications include anastomotic stricture (AS) and non-anastomotic stricture (NAS); AS can often be managed with endoscopic retrograde cholangiopancreatography (ERCP) combined with stent placement. For NAS, studies suggest that hypothermic oxygenated perfusion and normothermic machine perfusion can reduce ischemia-reperfusion injury, thereby decreasing the incidence of NAS (20, 65).

For extrahepatic biliary strictures, due to favorable anatomical accessibility, HJ is currently the first-line surgical treatment for iatrogenic extrahepatic biliary strictures, achieving long-term biliary patency rates of 80%–90% (66). PTBD has demonstrated high safety, a low complication rate, and significant improvements in liver function recovery when used to relieve obstructive jaundice caused by extrahepatic malignancies (67). Additionally, magnetic compression anastomosis combined with stent placement has shown promising outcomes in treating complete extrahepatic biliary obstruction, effectively alleviating biliary strictures (68).

To further reduce the risk of biliary injuries, intraoperative auxiliary techniques have gained significant attention. For example, during cholecystectomy, intravenous injection of indocyanine green combined with near-infrared fluorescence imaging allows clear visualization of the biliary anatomy, reducing the likelihood of inadvertent bile duct injury (69). In summary, various surgical approaches offer patients with biliary stricture diverse options, while the introduction of new adjunctive technologies and optimized techniques provides renewed hope for improved outcomes and surgical success.

### 4.2 Non-surgical treatment

Non-surgical treatment primarily focuses on strategies such as immune modulation, bile acid regulation, and gut microbiota modulation to alleviate biliary inflammation and slow the progression of fibrosis. Among these, immune modulation demonstrates clear advantages in the treatment of immune-mediated biliary strictures; bile acid regulation plays a critical role in managing secondary cholestasis and biliary narrowing; and modulation of gut microbiota is particularly effective for infectious biliary strictures. With increasing research into the biliary microenvironment, its therapeutic potential is also expanding to other etiologies of biliary strictures.

In terms of immune modulation, NF-κB is a key therapeutic target of great interest in the regulation of biliary injury, fibrosis, and stricture, and strategies targeting this pathway are actively being investigated (70). Furthermore, Jongthawin et al. (71) found that COX-2 inhibitors may exhibit potential antitumor effects in cholangiocarcinoma by inhibiting PGE2 production and cell migration. Stoelinga et al. (72) demonstrated that immunosuppressants combined with ursodeoxycholic acid (UDCA) effectively alleviate immune-mediated biliary injury. Additionally, Joo et al. (73) reported that corticosteroids combined with tissue-engineered stents can significantly improve fibrosis and inflammation in benign biliary strictures.

In bile acid regulation, UDCA remains the first-line therapy for immune-mediated cholangitis, as it reduces bile toxicity, stabilizes cell membranes, and suppresses inflammatory responses. Combination therapy of UDCA with FXR or PPAR agonists may further enhance efficacy, as these agents activate bile acid metabolism-related receptors (FXR, PPAR), inhibit CYP7A1 expression, and synergistically reduce cholestasis (74).

For gut microbiota modulation, balancing the intestinal microbiota can reduce harmful bile acid metabolites and their damaging effects on the bile duct by regulating the gut-liver axis. In treating gut-derived biliary infections, antibiotics (e.g., β-lactams, cephalosporins, fluoroquinolones, and carbapenems) combined with biliary drainage and decompression effectively eradicate infectious agents, relieve intrabiliary pressure, and prevent further injury and stricture formation (75). Studies have also shown that fecal microbiota transplantation demonstrates potential in the treatment of PSC, as increased microbial diversity and abundance are closely associated with improvements in ALP levels (76). Similarly, vancomycin may alleviate PSC by modulating gut microbiota and immune responses (77).

In summary, non-surgical treatments, through multi-dimensional and multi-targeted strategies, can effectively reduce inflammation and fibrosis associated with biliary injuries. With ongoing research, these therapeutic approaches are expected to be further refined, opening new directions and prospects for the management of biliary stricture and injury.

### 4.3 Novel therapeutic approaches

Currently, emerging treatment modalities such as targeted therapy, gene therapy, regenerative replacement therapies, and tissue-engineered biliary stent implantation have garnered significant attention in the field of biliary injury repair, demonstrating considerable potential and promising prospects for future applications.

#### 4.3.1 Targeted therapy

Current research increasingly focuses on the regulation of key signaling pathways involved in the pathological processes of bile duct injury repair. Transforming growth factor-β (TGF-β) is a pivotal cytokine mediating hepatic fibrosis, contributing to excessive ECM deposition and ultimately fibrosis. Xu et al. (78) demonstrated in a bile duct ligation (BDL) mouse model that combined treatment with pirfenidone and andrographolide attenuates biliary fibrosis by modulating the TGF-β/Smad signaling pathway and inhibiting hepatic stellate cell activation. Additionally, other studies have shown that ECM1 and integrins play significant roles in bile duct fibrosis by regulating TGF-β activation (79, 80). Dysregulation of the Wnt signaling pathway is closely associated with bile duct injury, inflammation, and fibrosis. Ayers et al. (81) found in a BDL mouse model that inhibiting the Wnt signaling pathway effectively reduces abnormal cholangiocyte activation and disrupts the nuclear factor-κB-dependent inflammatory axis, thereby mitigating cholestatic injury. The Notch signaling pathway, a highly conserved signaling network, profoundly impacts the pathophysiological processes of bile duct injury repair. Huang et al. (82) demonstrated in a BDL mouse model that reverse transcriptase reduces bile duct formation associated with the Dlk1/Notch/Sox9 signaling axis and significantly alleviates bile duct proliferation and fibrosis caused by cholestasis. Furthermore, Xu et al. showed that bone marrow mesenchymal stem cells overexpressing Numb, a negative regulator of the Notch signaling pathway, might represent a novel strategy for treating cholestatic liver cirrhosis (83).

In conclusion, in-depth investigation of critical signaling pathways involved in bile duct injury repair provides vital insights into its molecular mechanisms and facilitates the development of precise therapeutic strategies. Future research will emphasize integrating these regulatory mechanisms and translating them into clinical applications.

#### 4.3.2 Gene therapy

Gene editing technologies have shown remarkable potential in the treatment of hereditary biliary stricture-related diseases. For instance, in CF, CRISPR-mediated correction of CFTR gene mutations has demonstrated therapeutic potential in both cell and organoid models. One study successfully corrected CF-related L227R and N1303K mutations using CRISPR technology, resulting in restored CFTR protein glycosylation, localization, and function in HEK293T and 16HBE cell lines, as well as in patient-derived human nasal epithelial cells and rectal organoids. This research not only underscores the high specificity and feasibility of gene editing in CF therapy but also suggests its translational potential for future liver–biliary system CF models (84). Moreover, Biliary organoid models have been reported to be used for investigating disease mechanisms and evaluating gene therapy approaches. For example, organoids derived from induced pluripotent stem cells (iPSCs) with Alagille syndrome have been established to dissect the bile duct developmental defects caused by Jagged1/Notch2 signaling abnormalities, providing an experimental foundation for future personalized cell transplantation or combined gene-cell therapies (85).

The development of safe and efficient gene delivery systems is a critical step toward clinical translation. Adeno-associated virus (AAV) vectors, due to their low immunogenicity and high targeting efficiency, have become increasingly favored for liver-directed gene delivery. Larrey et al. (86) discussed challenges associated with AAV and other vectors in the context of gene therapy-related drug-induced liver injury, offering valuable insights for their future application in biliary diseases. Additionally, nanoparticles are being explored as carriers to achieve targeted delivery of gene-editing tools to cholangiocarcinoma cells, potentially reducing the risk of systemic immune responses (87). In summary, both the development of gene-editing technologies and the advancement of delivery vectors are accelerating the translation of gene therapy into the field of biliary diseases. Future research should focus on improving the safety and efficiency of in vivo gene editing and exploring its therapeutic potential for biliary stricture.

#### 4.3.3 Regenerative replacement therapy

Stem cell and organoid technologies provide important avenues for cell and tissue regeneration following biliary injury. In addition to their multi-lineage differentiation potential, stem cells possess multiple paracrine effects, including anti-inflammatory, immunomodulatory, pro-angiogenic, and anti-apoptotic activities, which allow them to modulate the biliary repair microenvironment at multiple levels. Mesenchymal stem cells (MSCs) are widely sourced from bone marrow, placenta, umbilical cord, liver, adipose tissue, and other adult or perinatal tissues. In biliary injury repair research, MSCs contribute through: (1) differentiation into hepatocyte-like or biliary-like cells to replenish cellular loss; (2) secretion of soluble factors and growth factors (e.g., HGF, IL-10, TGF-β modulators) to suppress inflammation and fibrosis; and (3) release of extracellular vesicles that deliver microRNAs, proteins, and lipid signals to promote biliary epithelial regeneration and attenuate fibrotic deposition (88).

Organoids are three-dimensional structured models derived from expanded stem cells or specific biliary epithelial cells, capable of partially recapitulating the spatial polarity, transport functions, and molecular phenotypes of native biliary tissue. They serve as both a cell source for regenerative repair and an experimental platform for mechanistic studies and drug screening (85). Sampaziotis et al. (89) were the first to report the feasibility of using human-derived biliary epithelial organoids for bile duct repair, laying the conceptual foundation for biliary regenerative medicine. Research has shown that biliary organoids can effectively mimic disease models such as hepatic ischemia-reperfusion injury and BA, demonstrating great potential for studying the mechanisms of biliary injury and its therapeutic strategies (85). Beyond disease modeling, biliary organoids are also valuable for investigating absorptive-secretory functions of the biliary epithelium, immune modulation, and cross-talk with stromal and immune cells, thereby providing an experimental platform for the development of novel precision therapies.

#### 4.3.4 Tissue-engineered stents

ERCP combined with stent implantation is currently a widely used approach for repairing biliary injuries and strictures as well as preventing and treating fibrosis, particularly in cases of iatrogenic or traumatic bile duct injury. Traditional biliary stents offer good patency and wide applicability; however, they are inevitably associated with high recurrence rates of stricture and severe inflammatory responses. In contrast, tissue-engineered stents, owing to their excellent biocompatibility and tissue regeneration capability, have shown tremendous potential in the field of biliary stricture management. In recent years, the design and fabrication of biliary stents have rapidly advanced, with functionalization and personalization strategies significantly improving clinical outcomes in the treatment of biliary strictures (90). For example, Lee et al. (91) developed a biodegradable polymer stent with drug-release properties that can continuously deliver anti-inflammatory agents during gradual degradation, thereby effectively suppressing postoperative inflammation and excessive tissue repair. This stent demonstrated notable anti-inflammatory and anti-restenosis effects in a porcine model, making it particularly suitable for patients undergoing liver transplantation or those with chronic biliary diseases. Similarly, Nagakawa et al. (92) designed a self-expanding biliary stent composed of high-strength hydrogel, which exhibited excellent endoscopic delivery performance and favorable biomechanical stability in animal experiments, laying an important foundation for future clinical research.

Meanwhile, the integration of 3D printing technology with advanced biomaterials has provided new avenues for the personalized design of biliary stents. Through precise structural control and customized design, it is possible to maintain the mechanical strength of the stent while improving its compatibility with biliary tissue. For instance, Mazari-Arrighi et al. (93) successfully encapsulated rat bile duct cells within a 3D-printed scaffold to construct a branched network structure resembling the native bile duct, establishing a solid foundation for future biliary tree reconstruction and personalized regenerative therapies. Additionally, Lee et al. (94) employed 3D printing to fabricate a functionalized biodegradable stent with a nano-engineered surface, which effectively supported the bile duct and exhibited excellent mechanical properties and biocompatibility in a rabbit model.

Despite remarkable progress in biomimetic design and functional optimization, tissue-engineered stents still face challenges in maintaining cell viability, optimizing material mechanical strength, controlling immune responses, minimizing infection risk, and standardizing 3D printing protocols. Future research should continue to explore and overcome these obstacles to facilitate the widespread clinical application of these novel stents.

## 5 Conclusions and perspectives

Biliary stricture is a complex pathological condition triggered by multiple factors, with its onset and progression closely associated with biliary epithelial injury, inflammatory responses, and fibrotic ECM deposition. This review summarizes the major types and pathogenic mechanisms of biliary stricture, as well as recent advances in treatment strategies ranging from traditional surgical interventions to non-surgical approaches, and emerging therapies including targeted therapy, gene editing, regenerative replacement therapies, and tissue-engineered stents.

Although current treatment modalities for biliary stricture have expanded beyond traditional surgical interventions to include non-surgical and innovative technologies, significant challenges remain. First, as biliary stricture is a disease with diverse etiologies and complex mechanisms, treatment strategies vary considerably across etiologies. Developing a unified framework to guide therapeutic development and clinical management remains a major challenge. It is noteworthy that different etiologies of biliary injury seem to share similar pathological processes, including epithelial regeneration, inflammatory responses, and fibrotic deposition, all of which involve multiple key signaling pathways. This shared pathological mechanism not only offers a new perspective on the etiological complexity of biliary stricture but also lays a theoretical foundation for precision therapies targeting these signaling pathways. In the future, interventions focused on these core pathological nodes may represent critical breakthroughs in biliary stricture treatment.

Meanwhile, emerging therapeutic strategies such as gene editing, organoids, and tissue-engineered stents are evolving and showing great potential. However, off-target effects, delivery efficiency, and long-term safety remain key bottlenecks to their clinical application. Improving the precision and efficiency of gene-editing tools will be a primary focus for future research (95). regenerative replacement therapies, as cutting-edge approaches in regenerative medicine, have achieved significant progress in disease modeling and drug screening, yet their application in actual organ replacement and functional reconstruction is still at an early exploratory stage. Tissue-engineered stents have shown promising results in the repair of extrahepatic biliary strictures; however, given the structural complexity of the intrahepatic biliary network, current stent designs and implantation techniques remain insufficient. Developing implantable stents that accurately replicate intrahepatic bile duct structures and achieve long-term functional integration will be a key research focus in the field of tissue engineering.

Importantly, traditional research often classifies and treats biliary strictures based on their etiology while neglecting the shared pathological processes underlying different stricture types. In the future, integrating this common pathological mechanism into combination therapies–incorporating signaling pathway modulation, immune intervention, and regenerative repair–may maximize therapeutic efficacy. With the convergence and collaborative development of targeted therapy, gene editing, regenerative replacement therapies, and tissue-engineered technologies, the translation from basic research to clinical applications is expected to accelerate, optimizing personalized strategies for biliary injury repair and ultimately providing patients with safer, more precise, and more effective treatment options.
